# History, Virology, Pathogenesis, Epidemiology, and Laboratory Diagnosis of Dengue Virus: A Focused Review

**DOI:** 10.7759/cureus.109151

**Published:** 2026-05-18

**Authors:** Ravindra V Shinde, Shivaji T Mohite, Pratik P Durgawale, Satish R Patil, Anjali R Shinde, Sandeep B Shinde

**Affiliations:** 1 Department of Microbiology, Krishna Institute of Medical Sciences, Krishna Vishwa Vidyapeeth (Deemed to be University), Karad, IND; 2 Department of Molecular Biology and Genetics, Krishna Institute of Medical Sciences, Krishna Vishwa Vidyapeeth (Deemed to be University), Karad, IND; 3 Department of Pharmacology, Krishna Institute of Medical Sciences, Krishna Vishwa Vidyapeeth (Deemed to be University), Karad, IND; 4 Department of Musculoskeletal Sciences, Krishna College of Physiotherapy, Krishna Vishwa Vidyapeeth (Deemed to be University), Karad, IND

**Keywords:** arboviral infections, dengue epidemiology, dengue fever, dengue pathogenesis, dengue virus, flavivirus, igm elisa, laboratory diagnosis of dengue, ns1 antigen

## Abstract

Dengue virus (DENV) is a mosquito-borne flavivirus with four antigenically distinct serotypes and represents a leading cause of acute febrile illness in tropical and subtropical regions worldwide. This focused review summarizes the historical evolution of dengue disease descriptions, virus discovery, vectors and transmission dynamics, viral structure, mechanisms of pathogenesis-including antibody-dependent enhancement (ADE) and non-structural protein-1 (NS1)-mediated endothelial dysfunction-clinical classification, current epidemiology with emphasis on India, and laboratory diagnostic approaches. We highlight persistent gaps in molecular surveillance, particularly incomplete genotype and lineage characterization in routine hospital-based studies, and discuss emerging diagnostic and surveillance tools such as real-time reverse transcription polymerase chain reaction (RT-PCR), NS1 antigen detection, isothermal amplification, plaque reduction neutralization tests (PRNT), and genomic sequencing. The review concludes by outlining priorities for strengthening surveillance, diagnostics, and research to improve clinical management, vaccine policy, and public health response in endemic and peri-urban/rural settings.

## Introduction and background

Descriptions of dengue-like febrile illness date back several centuries, with early reports originating from Asia, Africa, and the Americas during the 17th and 18th centuries. These illnesses were historically described using terms such as “break-bone fever,” “kidinga pepo,” and “dandy fever,” reflecting the characteristic severe myalgia, arthralgia, and postural stiffness associated with the disease [1,2]. The occurrence of widespread epidemics during this period coincided with the expansion of maritime trade, which facilitated the dissemination of *Aedes aegypti* mosquitoes through shipping routes and contributed to the geographic spread of dengue transmission [3]. Recognition of dengue as a mosquito-borne viral disease emerged in the early 20th century following experimental confirmation of transmission by *Aedes* mosquitoes [3,4]. Dengue virus (DENV) was first isolated independently in Japan and Hawaii during the 1940s, establishing its viral etiology and enabling its classification within the genus *Flavivirus* [4]. By the mid-20th century, four antigenically distinct serotypes (DENV-1 to DENV-4) were identified, providing important insights into dengue immunopathogenesis and the increased risk of severe disease during secondary heterologous infections [5,6].

Over the past five decades, dengue epidemiology has shifted from sporadic urban outbreaks to sustained hyperendemic transmission in many tropical and subtropical regions [7]. Rapid urbanization, population growth, increased global travel, climate change, and weakening vector control programs have collectively contributed to the expansion of dengue transmission, placing nearly 3.9 billion people at risk worldwide [2,8-10]. Consequently, the World Health Organization (WHO) recognizes dengue as one of the fastest-spreading vector-borne viral diseases and a major global public health challenge [1,10].

In India, dengue has emerged as a major public health concern over recent decades, with a marked increase in both the frequency and geographic distribution of outbreaks [11-13]. Historically confined to urban centers, dengue transmission has progressively expanded into peri-urban and rural regions, reflecting changing ecological and demographic patterns [11]. All four DENV serotypes (DENV-1 to DENV-4) are known to co-circulate in different parts of the country, contributing to hyperendemic transmission and increasing the risk of severe dengue during secondary infections [13,14]. Periodic shifts in dominant circulating serotypes and genotypes have been reported across multiple states and are often associated with variations in outbreak magnitude and disease severity [15-18]. Seasonal transmission typically peaks during and after the monsoon months due to increased vector breeding, although year-round transmission has been documented in several endemic regions [11,19]. Rapid urbanization, high population density, inadequate water management, climate variability, and increased human mobility have further facilitated the persistence and expansion of dengue transmission in India [12,19].

Given the expanding geographic distribution of dengue and the co-circulation of multiple serotypes across India, timely and accurate laboratory diagnosis plays a critical role in clinical management, outbreak detection, and implementation of effective public health interventions [20-22]. The overlapping clinical presentation of dengue with other acute febrile illnesses commonly encountered in endemic settings further underscores the importance of reliable diagnostic approaches for early case confirmation [20,22]. In addition, continuous laboratory-supported surveillance is essential for monitoring circulating serotypes and genotypes, detecting shifts in transmission dynamics, and guiding vector control and prevention strategies [14,18]. Therefore, the integration of clinical assessment with molecular, antigen-based, and serological diagnostic methods forms the cornerstone of effective dengue surveillance systems, particularly in resource-constrained and high-burden regions such as India [21-23]. Strengthening laboratory capacity and harmonizing diagnostic algorithms within national surveillance frameworks remain key priorities for improving dengue control and outbreak preparedness [23].

## Review

Search strategy

**Figure 1:**
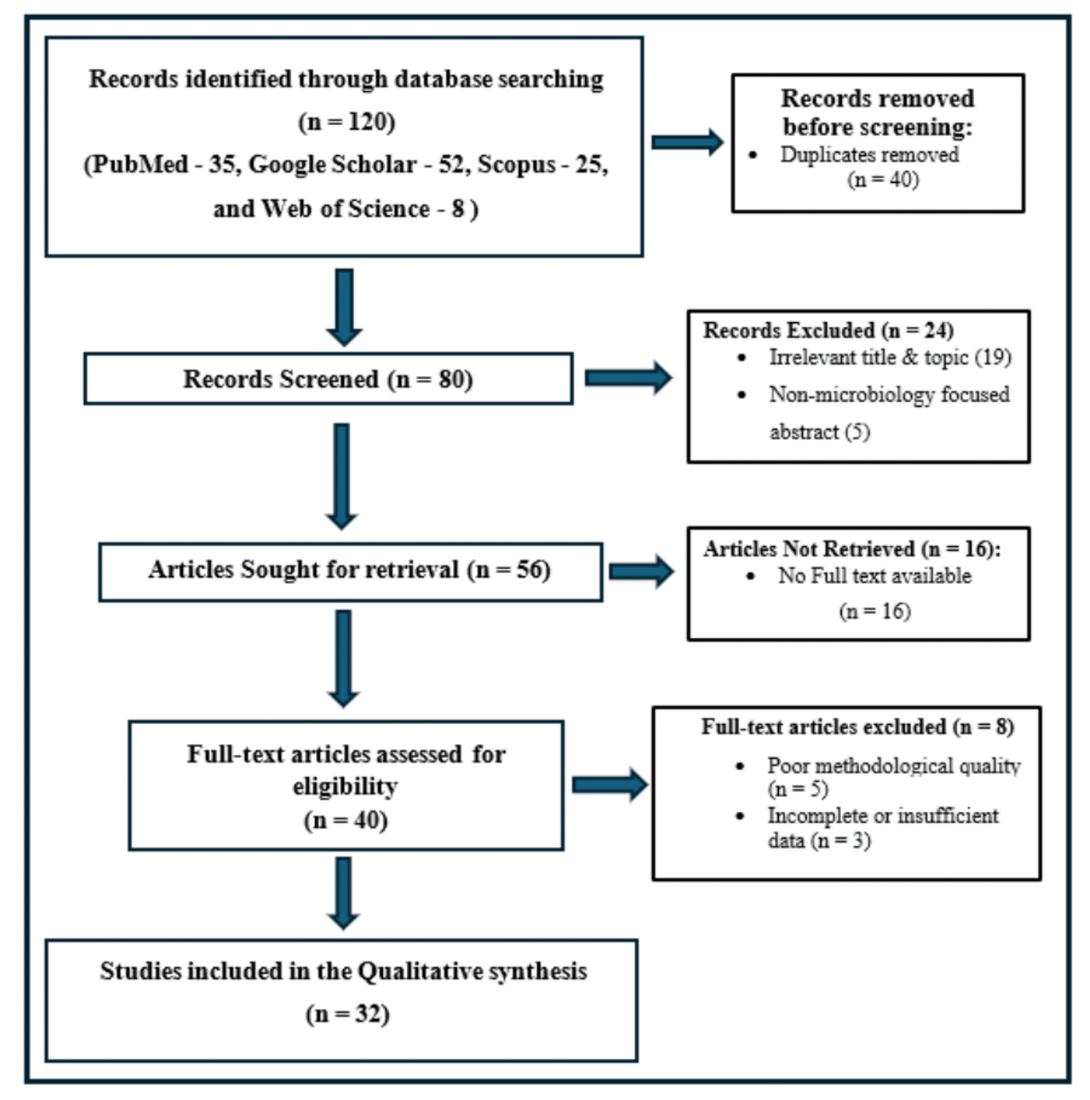
PRISMA-style flow diagram showing the study selection process for the narrative review n: number of articles

A structured literature search was conducted to identify relevant articles on the history, virology, pathogenesis, epidemiology, and laboratory diagnosis of DENV infection. Electronic databases, including PubMed, Google Scholar, Scopus, and Web of Science, were systematically searched for peer-reviewed literature published up to December 2025. The search strategy incorporated Medical Subject Headings (MeSH) terms and keywords such as “Dengue”, “Dengue Virus”, “Flavivirus”, “Pathogenesis”, “Epidemiology”, “Laboratory Diagnosis”, “NS1 antigen”, and “RT-PCR”, combined using appropriate Boolean operators (AND/OR). Figure 1 presents the PRISMA-style flow diagram illustrating the study selection process. A total of 120 records were identified through database searching. After removal of duplicates (n = 40), 80 records were screened based on titles and abstracts. Of these, 24 records were excluded due to irrelevant topics (n = 19) and non-microbiology-focused abstracts (n = 5). The remaining 56 articles were sought for retrieval; however, 16 could not be retrieved due to the unavailability of full texts. Consequently, 40 full-text articles were assessed for eligibility. Eligibility standards were predefined and applied during screening based on relevance to the topic, microbiology-focused content, availability of full text, methodological quality, and adequacy of data for analysis. Methodological quality and risk of bias assessment were performed using the SANRA (Scale for the Assessment of Narrative Review Articles) tool, a validated six-item scale specifically designed for evaluating narrative reviews [24]. Of the 40 full-text articles assessed, eight were excluded due to poor methodological quality based on SANRA assessment (n = 5) and incomplete or insufficient data (n = 3). Ultimately, 32 studies met the inclusion criteria and were included in the qualitative synthesis of this narrative review.

DENV: taxonomy, structure, and serotypes

DENV belongs to the genus *Flavivirus* within the family Flaviviridae, which also includes several medically important arboviruses such as Zika virus, West Nile virus, Japanese encephalitis virus, and yellow fever virus [1]. Members of this genus share common structural and genomic characteristics, including an enveloped virion architecture and a positive-sense single-stranded ribonucleic acid (RNA) genome, enabling efficient replication in both vertebrate hosts and arthropod vectors. The ability of DENV to maintain transmission cycles between humans and *Aedes* mosquitoes, particularly *A. aegypti* and *Aedes albopictus*, contributes to its widespread distribution across tropical and subtropical regions.

DENV is an enveloped, positive-sense, single-stranded RNA virus with a genome of approximately 10.6-11 kb encoding a single open reading frame flanked by untranslated regions that regulate viral translation and replication [2,3]. The genomic RNA functions directly as messenger RNA upon entry into host cells and is translated into a large polyprotein that undergoes co- and post-translational cleavage by both host and viral proteases. This processing results in the formation of three structural proteins-capsid (C), precursor membrane/membrane (prM/M), and envelope (E)-and seven non-structural proteins (NS1-NS5), each of which plays a critical role in viral assembly, replication, and host interaction [4,6].

The C protein associates with viral RNA to form the nucleocapsid core, while the prM protein acts as a chaperone during virion maturation by preventing premature conformational rearrangements of the E glycoprotein during intracellular transport through the secretory pathway. Cleavage of prM into the mature M protein by host furin proteases within the trans-Golgi network is essential for generating infectious virions [4,5]. The E glycoprotein, which forms homodimers on the virion surface, mediates receptor binding, viral attachment, and membrane fusion during host-cell entry and represents the principal target of neutralizing antibodies elicited during infection. Structural rearrangements of the E protein under acidic endosomal conditions facilitate fusion between viral and host membranes, enabling release of viral RNA into the cytoplasm and initiation of replication [4,5].

**Figure 2:**
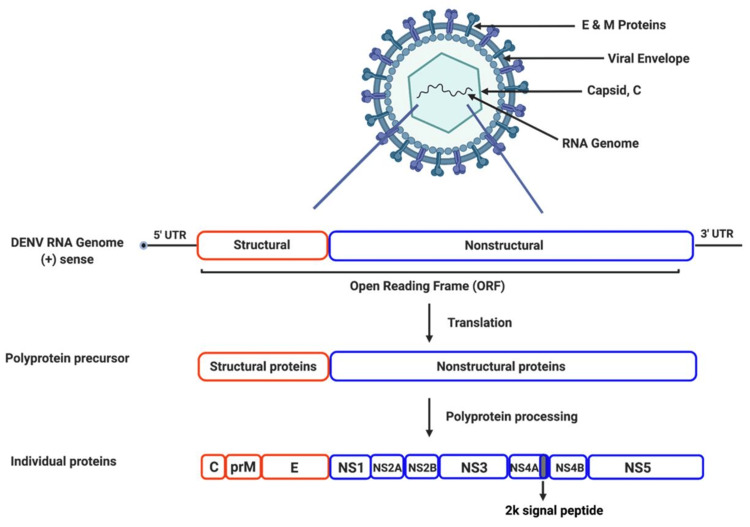
Schematic representation of the dengue virus (DENV) virion and genome organization C: capsid; M: membrane (part of E-M proteins); E: envelope; NS1-NS5: non-structural proteins 1-5; RNA: ribonucleic acid; UTR: untranslated region Image sourced from Obi et al. [8] licensed under Creative Commons Attribution 4.0 International (CC BY 4.0).

The non-structural proteins perform multiple regulatory and enzymatic functions essential for viral replication and immune evasion. NS1 is a multifunctional glycoprotein that exists in membrane-associated and secreted forms and plays an important role in viral replication complex formation, complement activation, immune modulation, and endothelial dysfunction associated with severe dengue manifestations [6,7]. NS2A and NS2B participate in replication complex assembly, with NS2B acting as a cofactor for the NS3 protease. NS3 possesses both serine protease and helicase activities required for polyprotein processing and RNA unwinding, respectively, while NS4A and NS4B contribute to remodeling of host intracellular membranes to create replication compartments. NS5, the largest and most conserved dengue viral protein, functions as the RNA-dependent RNA polymerase and methyltransferase responsible for genome replication and RNA capping, and also interferes with host interferon signaling pathways to facilitate viral persistence within infected cells [6,7]. Figure 2 shows a schematic representation of DENV virion and genome organization, showing structural (C, prM/M, and E) and non-structural (NS1-NS5) proteins encoded within the single-stranded positive-sense RNA genome [8].

Four antigenically distinct serotypes of DENV (DENV-1 to DENV-4) circulate globally, each capable of causing the full spectrum of clinical disease ranging from asymptomatic infection to severe dengue with plasma leakage and shock. Infection with one serotype confers long-lasting homotypic immunity; however, cross-protective immunity against heterologous serotypes is temporary and incomplete. Subsequent infection with a different serotype is associated with an increased risk of severe disease through antibody-dependent enhancement (ADE), a phenomenon in which non-neutralizing or subneutralizing antibodies facilitate increased viral entry into Fc receptor-bearing cells such as monocytes and macrophages, resulting in higher viral replication and exaggerated immune activation [9-11]. This immunopathological mechanism represents a central challenge in dengue vaccine development and contributes significantly to the complexity of dengue epidemiology in hyperendemic regions where multiple serotypes co-circulate simultaneously. The co-circulation of all four serotypes, together with ongoing genetic evolution and lineage replacement within each serotype, has important implications for disease severity patterns, outbreak dynamics, and long-term immunity at the population level. Therefore, understanding the taxonomy, structural organization, and serotype diversity of DENV provides a critical foundation for interpreting transmission trends, improving diagnostic strategies, and guiding vaccine and surveillance efforts in endemic settings [11,12].

Vectors and transmission cycle

DENV is transmitted primarily by *A. aegypti*, the principal urban vector, and *A. albopictus*, an ecologically adaptable secondary vector that contributes to transmission across a broader range of environmental settings [1,2]. *A. aegypti* is highly anthropophilic and endophilic, preferentially feeding on humans and resting indoors, which enhances its efficiency as a vector in densely populated urban environments. It commonly breeds in artificial water containers such as household storage vessels, discarded tires, flower pots, and construction sites, facilitating sustained transmission in rapidly urbanizing regions with inadequate water management and sanitation infrastructure [3,4]. In contrast, *A. albopictus* demonstrates greater ecological flexibility and can utilize both artificial and natural breeding habitats, including tree holes and leaf axils, thereby extending dengue transmission into peri-urban and rural environments and contributing to the geographic expansion of the disease [5,6]. Transmission begins when a female mosquito ingests viremic blood from an infected individual during a blood meal. Following ingestion, the virus infects and replicates within the midgut epithelium of the mosquito, subsequently disseminates through the hemocoel, and reaches the salivary glands after completion of the extrinsic incubation period. Once the salivary glands are infected, the mosquito becomes capable of transmitting the virus to susceptible individuals during subsequent feeding cycles, thereby sustaining human-mosquito-human transmission in endemic settings [8-10]. Environmental factors such as temperature, rainfall, and humidity play a critical role in influencing mosquito breeding patterns, survival rates, biting behavior, and viral replication within the vector, collectively determining vector abundance and overall vectorial capacity. These climatic determinants strongly influence seasonal transmission dynamics and are closely associated with the occurrence of dengue outbreaks, particularly during and following monsoon periods in endemic regions such as India [3,11-15].

Pathogenesis and determinants of severity

**Figure 3:**
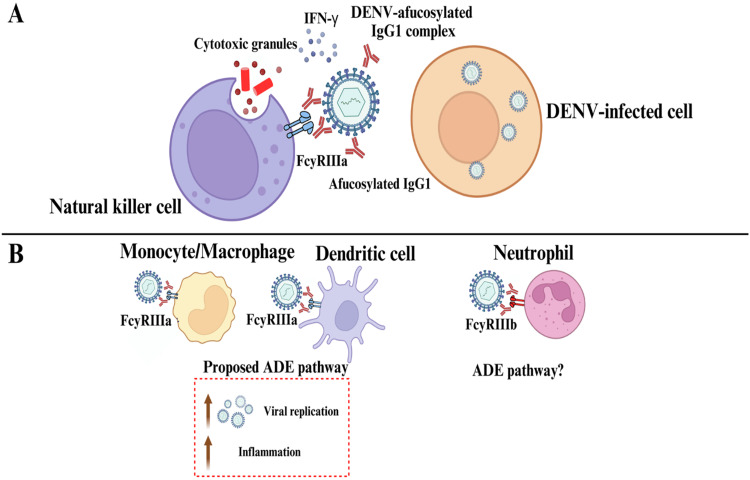
Immune mechanisms involved in dengue virus (DENV) infection and antibody-dependent enhancement (ADE) (A) NK cell-mediated antibody-dependent cellular cytotoxicity against DENV-infected cells via FcγRIIIa interaction, resulting in the release of cytotoxic granules and IFN-γ; (B) ADE pathway promoting DENV entry, replication, and inflammation in immune cells. IgG1: immunoglobulin G1; FcγRIIIa/FcγRIIIb: Fc gamma receptor IIIa/IIIb; IFN-γ: interferon-gamma; NK cell: natural killer cell; ADCC: antibody-dependent cellular cytotoxicity Reproduced from Teo et al. [16] under Creative Commons Attribution 4.0 International (CC BY 4.0).

Dengue pathogenesis is multifactorial and results from complex interactions between viral virulence factors, host immune responses, and genetic susceptibility, which together determine the clinical spectrum ranging from asymptomatic infection to severe dengue with plasma leakage and shock [1-7]. Following transmission through the bite of an infected *Aedes* mosquito, DENV initially infects dendritic cells, monocytes, macrophages, and endothelial cells at the site of inoculation and subsequently disseminates through lymphatic and hematogenous routes. Viral replication within these target cells contributes to viremia and triggers activation of both innate and adaptive immune responses, including the production of interferons, pro-inflammatory cytokines, and chemokines that influence disease progression and severity [1-7]. A key mechanism implicated in severe dengue is ADE, which occurs during secondary infection with a heterologous serotype. In this process, pre-existing non-neutralizing or subneutralizing antibodies facilitate enhanced viral entry into Fcγ receptor-bearing cells such as monocytes and macrophages, resulting in increased viral replication and higher circulating viral loads. This amplified infection promotes exaggerated immune activation characterized by increased cytokine production, complement activation, and endothelial dysfunction, thereby contributing to plasma leakage and severe clinical manifestations as depicted in Figure 3 [16].

In addition to ADE-mediated mechanisms, the DENV NS1 plays an independent role in disease severity through direct pathogenic effects on the vascular endothelium. Circulating NS1 disrupts endothelial glycocalyx integrity, activates complement pathways, and triggers Toll-like receptor 4 (TLR4)-mediated inflammatory signaling cascades, leading to increased vascular permeability and contributing to hallmark features of severe dengue such as hemoconcentration and shock [13-15]. Furthermore, NS1-mediated immune activation enhances cytokine release and promotes interactions between immune cells and endothelial surfaces, amplifying vascular injury during critical phases of infection. Host-related determinants also significantly influence dengue severity. Clinical outcomes vary according to age, with children and elderly individuals often demonstrating increased vulnerability to severe disease manifestations. Comorbid conditions such as diabetes mellitus, hypertension, chronic kidney disease, and obesity have been associated with an increased risk of complications. Nutritional status, particularly micronutrient deficiencies, may further impair immune regulation and influence disease progression. In addition, genetic susceptibility plays an important role, with polymorphisms involving human leukocyte antigen (HLA) alleles, cytokine genes, and Fcγ receptor variants contributing to interindividual variability in immune responses and clinical outcomes during dengue infection [17-23,25,26]. Collectively, these interacting viral and host determinants contribute to the development of vascular permeability, thrombocytopenia, coagulopathy, and systemic inflammatory responses that characterize severe dengue [18-23,25,26]. A comprehensive understanding of these pathogenic mechanisms is essential for improving risk stratification, guiding therapeutic interventions, and informing vaccine development strategies in dengue-endemic regions. Severe dengue is also characterized by complex immunopathological responses involving complement activation, cytokine release, and T-cell-mediated immune responses that contribute to endothelial dysfunction and plasma leakage during the critical phase of illness [16-18]. The interaction between antibodies, monocytes/macrophages, T lymphocytes, and inflammatory mediators leads to capillary leak syndrome.

Clinical presentation and WHO classification

Clinical manifestations of dengue infection range widely from asymptomatic infection or mild undifferentiated febrile illness to severe dengue characterized by plasma leakage, severe bleeding, or organ dysfunction [1-6]. After an incubation period of approximately 4-10 days following the infective mosquito bite, patients typically present with an abrupt onset of high-grade fever accompanied by headache, retro-orbital pain, myalgia, arthralgia, nausea, vomiting, rash, and leukopenia. These symptoms constitute the classical presentation of dengue fever and are often described as “break-bone fever” because of the severity of musculoskeletal pain. A transient maculopapular or erythematous rash may appear during the febrile phase or early recovery phase, and mild hemorrhagic manifestations such as petechiae, epistaxis, or gum bleeding may also occur in some patients [1-4]. The clinical course of dengue infection typically progresses through three recognized phases: the febrile phase, the critical phase, and the recovery phase. The febrile phase is characterized by high fever and generalized constitutional symptoms lasting two to seven days. As fever subsides, some patients enter the critical phase, during which increased capillary permeability may lead to plasma leakage, hemoconcentration, pleural effusion, ascites, and circulatory compromise. This phase is particularly important for early recognition because timely clinical monitoring and fluid management can prevent progression to shock and severe complications. The recovery phase is marked by gradual reabsorption of extravascular fluid, stabilization of hemodynamic status, and improvement in clinical symptoms, often accompanied by a characteristic convalescent rash and transient bradycardia [1-7].

The 2009 WHO classification categorizes dengue into dengue without warning signs, dengue with warning signs, and severe dengue, improving clinical triage, case management, and surveillance compared with the earlier 1997 classification system [3,10,11]. The National Vector-Borne Disease Control Programme (NVBDCP) 2023 guidelines similarly adopt a standardized framework for the diagnostic and clinical classification approach to dengue in India. Warning signs that indicate possible progression to severe disease include persistent vomiting, severe abdominal pain, mucosal bleeding, clinical fluid accumulation, lethargy or restlessness, hepatomegaly, and a rising hematocrit associated with rapidly falling platelet counts. Severe dengue is defined by the presence of severe plasma leakage leading to shock or respiratory distress, severe bleeding requiring clinical intervention, or severe organ involvement such as hepatitis, myocarditis, encephalopathy, or renal impairment. This classification framework supports early identification of high-risk patients and facilitates standardized reporting across surveillance systems [3,10-12]. Laboratory findings play an essential supportive role in diagnosis and prognostic assessment. Thrombocytopenia, progressive leukopenia, hemoconcentration due to plasma leakage, and elevated hepatic transaminase levels are common laboratory abnormalities observed during dengue infection. Rising hematocrit with falling platelet counts is particularly suggestive of impending severe disease and warrants close monitoring. Additional laboratory indicators such as prolonged coagulation parameters and metabolic abnormalities may be observed in complicated cases and help guide clinical decision-making regarding hospitalization and supportive management [19-23].

Laboratory diagnosis

Accurate laboratory diagnosis of DENV infection depends on appropriate sample collection, timing of specimen acquisition, and the phase of illness. While collecting blood samples for serological investigations, standard universal precautions should be followed. The day of onset of fever and the day of sample collection should be clearly mentioned, as these factors guide the selection of appropriate diagnostic tests. In general, NS1 antigen detection and molecular assays are preferred during the early viremic phase (days 1-5 of illness), whereas antibody-based assays are more useful after the fifth day of illness when humoral immune responses become detectable. In endemic regions, the early clinical manifestations of dengue often overlap with other febrile illnesses such as chikungunya, Zika virus disease, malaria, leptospirosis, typhoid fever, urinary tract infections, and other viral infections; therefore, laboratory confirmation is essential for accurate diagnosis and patient management [14,17,20].

During the acute phase of infection, detection of dengue NS1 antigen and viral RNA is the principal method for early diagnosis. NS1 is a highly conserved glycoprotein present in high concentrations in patient serum during the viremic stage and can be detected using ELISA-based assays with high specificity and sensitivity. Early identification of the NS1 antigen has important epidemiological significance because it facilitates prompt recognition of cases and implementation of control measures to limit transmission [14,17,20]. Molecular diagnostic techniques, particularly reverse transcription polymerase chain reaction (RT-PCR), nested RT-PCR, and real-time RT-PCR, have increasingly replaced conventional virus isolation methods as the standard approach for detecting DENV in acute-phase serum samples. These assays provide high sensitivity and specificity and also enable serotype identification, which is important for epidemiological surveillance and outbreak investigations [14,17,20].

Serological assays are mainly employed during the later stages of infection when viremia declines and antibody responses become detectable. IgM-capture enzyme-linked immunosorbent assay (MAC-ELISA) is widely used because it is simple, cost-effective, and does not require sophisticated laboratory infrastructure. Dengue-specific IgM antibodies generally become detectable around the fifth day of illness, although the timing may vary among individuals, with some patients developing detectable antibodies earlier or later in the course of infection. IgM antibodies may persist for several weeks to months and therefore indicate recent dengue infection [14,17,20]. IgG-ELISA is also used, primarily for differentiating primary and secondary dengue infections and for sero-epidemiological studies, although it is not considered definitive for acute diagnosis because IgG antibodies mainly reflect past exposure [14,17,20]. Other serological methods such as hemagglutination inhibition, complement fixation, and neutralization tests are available but are less commonly used because of technical limitations and complexity [14,17,20].

Isolation of DENV from clinical specimens such as acute-phase serum, plasma, buffy coat, autopsy tissues, or field-collected mosquitoes can be achieved when samples are obtained within the first five days of illness and processed promptly. However, virus isolation generally requires 7-10 days and is therefore of limited value for immediate clinical management [14,17,20]. Plaque reduction neutralization tests (PRNT) remain the reference standard for confirmation of serotype-specific neutralizing antibodies and are particularly important in vaccine evaluation and seroprevalence studies, although their application is largely restricted to specialized laboratories because of technical complexity [14,17,20].

Rapid diagnostic tests (RDTs) for NS1 antigen and dengue IgM/IgG antibodies are commercially available and can provide results within minutes. However, variability in sensitivity, specificity, and batch-to-batch performance has been reported, with some assays demonstrating high false-positive rates compared with standard laboratory methods. Consequently, their routine use for guiding clinical management is not universally recommended [14,17,20].

Integrated diagnostic approaches that combine molecular, antigen-based, and serological assays improve overall diagnostic sensitivity and specificity across different stages of infection. Such strategies are particularly important in dengue-endemic settings where co-circulation of multiple arboviruses can complicate clinical diagnosis [27-30]. In India, the National Center for Vector Borne Diseases Control (NCVBDC) has established a nationwide laboratory network comprising Sentinel Surveillance Hospitals and Apex Referral Laboratories to strengthen dengue surveillance and provide standardized diagnostic support. Standardization of diagnostic assays, use of validated reference reagents, and participation in external quality assurance programs are essential to ensure reliability, interlaboratory comparability, and consistency of surveillance data across regional and national networks [27-30].

Molecular epidemiology and genotype surveillance

Molecular epidemiology plays a critical role in understanding DENV evolution, transmission dynamics, and outbreak potential. Genotype surveillance provides valuable information on viral diversity, lineage replacement, and geographic spread of circulating strains, thereby supporting early detection of emerging variants associated with increased transmissibility or disease severity. Genotyping studies commonly target conserved genomic regions such as the C-prM, E, or NS5 regions, which are suitable for phylogenetic analysis and lineage classification, while whole-genome sequencing offers higher phylogenetic resolution and enables comprehensive characterization of viral genetic variation [1-4,20-23,25].

In India, co-circulation of multiple DENV serotypes and genotypes has been documented across different geographic regions, with periodic lineage replacement contributing to changing outbreak patterns and disease severity profiles. These observations highlight the importance of sustained genomic surveillance integrated with national vector-borne disease monitoring programs. Strengthening sequencing capacity and linking molecular epidemiological findings with clinical and entomological data will further enhance early outbreak detection and inform targeted public health interventions in endemic settings [19,26,31-33].

Dengue vaccines and diagnostic implications

Recent advances in dengue vaccine development represent an important milestone in disease prevention; however, vaccine deployment has important implications for diagnostic interpretation and surveillance strategies. Currently licensed dengue vaccines include CYD-TDV (Dengvaxia®) and TAK-003 (Qdenga®), both of which are live attenuated tetravalent vaccines designed to provide protection against all four DENV serotypes [8]. Safety concerns associated with Dengvaxia® in seronegative individuals necessitate pre-vaccination screening to determine baseline serostatus, as vaccination in previously unexposed individuals has been associated with an increased risk of severe disease following subsequent natural infection [16]. In contrast, TAK-003 (Qdenga®) has demonstrated broader efficacy across different serostatus groups and has shown promising protection against symptomatic dengue in clinical trials conducted in endemic populations [21-23,25]. The introduction of dengue vaccines complicates the interpretation of serological surveillance data because vaccine-induced antibodies may be difficult to distinguish from those generated during natural infection. Consequently, antigen-based assays such as NS1 detection and nucleic-acid-based diagnostic methods remain essential for the accurate identification of acute dengue infections in vaccinated populations. Integration of vaccination programs with strengthened laboratory surveillance systems is therefore critical for maintaining accurate disease burden estimates and evaluating vaccine impact over time.

Dengue in India: epidemiology and seroprevalence

India bears a substantial dengue burden with marked regional heterogeneity in transmission intensity, outbreak frequency, and serotype distribution. Among the four DENV serotypes, DENV-2 has been reported as the predominant circulating serotype in several regions of India and has frequently been associated with severe outbreaks and dengue hemorrhagic fever [31]. Seroprevalence estimates vary widely across the country, ranging from relatively low levels during interepidemic periods to more than 80% in hyperendemic urban settings where repeated exposure to multiple serotypes occurs over time. These variations reflect differences in vector density, climatic conditions, urbanization patterns, population mobility, and effectiveness of vector control programs across regions [3,12]. Hospital-based studies from rural Western Maharashtra have reported seroprevalence rates of approximately 10%, suggesting sustained low-level transmission and highlighting the possibility of underrecognized dengue burden in rural populations where access to diagnostic facilities remains limited. These findings underscore the importance of strengthening laboratory-supported surveillance systems in both urban and rural settings to improve early outbreak detection and guide targeted public health interventions.

Research gaps and future directions

Despite substantial progress in dengue surveillance and diagnostics, several important gaps remain in current understanding and control strategies. Limited genotype surveillance capacity in many regions restricts timely detection of emerging viral variants, while rural populations remain underrepresented in epidemiological studies despite increasing evidence of transmission beyond urban centers. In addition, although the Integrated Disease Surveillance Programme (IDSP) provides a structured surveillance platform, standardized molecular reporting and genotype surveillance remain limited in many tertiary care settings because of inadequate access to molecular diagnostic and sequencing facilities. Furthermore, insufficient sequencing infrastructure and variability in diagnostic assay availability continue to hinder comprehensive assessment of disease burden at national and regional levels. Future priorities should include expansion of genomic surveillance networks, strengthening of diagnostic infrastructure in resource-limited and rural settings, harmonization of laboratory and epidemiological reporting standards, and improved integration of molecular surveillance data with clinical, entomological, and vaccination program information. Such coordinated approaches will be essential for enhancing early outbreak detection, guiding vaccine deployment strategies, and improving vector control interventions aimed at reducing dengue transmission in endemic regions [2,3].

## Conclusions

Dengue remains an important and evolving public health challenge in India and other tropical regions due to rapid urbanization, climate variability, and changing vector ecology. Continuous circulation of multiple DENV serotypes highlights the need for strengthened laboratory-based surveillance. Integration of clinical, serological, molecular, and entomological monitoring is essential for early outbreak detection and effective disease control. Standardized diagnostic approaches and genomic surveillance can improve understanding of circulating strains and support timely public health interventions. Additionally, region-specific epidemiological data are critical for guiding vaccine policy and optimizing prevention strategies. Strengthening coordinated surveillance systems and vector control measures will play a key role in reducing dengue transmission and mitigating future outbreaks at both national and global levels.
